# Hybrid Topological Data Analysis and Deep Learning for Basal Cell Carcinoma Diagnosis

**DOI:** 10.1007/s10278-023-00924-8

**Published:** 2024-01-12

**Authors:** Akanksha Maurya, R. Joe Stanley, Norsang Lama, Anand K. Nambisan, Gehana Patel, Daniyal Saeed, Samantha Swinfard, Colin Smith, Sadhika Jagannathan, Jason R. Hagerty, William V. Stoecker

**Affiliations:** 1 Missouri University of Science &Technology, Rolla, MO 65209 USA; 2 University of Missouri, Columbia, MO USA; 3 A.T. Still, University of Health Sciences, Kirksville, MO USA; 4 University of Missouri, Kansas City Medical School, Kansas City, MO USA; 5 S&A Technologies, Rolla, MO USA

**Keywords:** Basal cell carcinoma, TDA, Persistent homology, Deep learning, Fusion, Telangiectasia, Transfer learning, Dermoscopy

## Abstract

A critical clinical indicator for basal cell carcinoma (BCC) is the presence of telangiectasia (narrow, arborizing blood vessels) within the skin lesions. Many skin cancer imaging processes today exploit deep learning (DL) models for diagnosis, segmentation of features, and feature analysis. To extend automated diagnosis, recent computational intelligence research has also explored the field of Topological Data Analysis (TDA), a branch of mathematics that uses topology to extract meaningful information from highly complex data. This study combines TDA and DL with ensemble learning to create a hybrid TDA-DL BCC diagnostic model. Persistence homology (a TDA technique) is implemented to extract topological features from automatically segmented telangiectasia as well as skin lesions, and DL features are generated by fine-tuning a pre-trained EfficientNet-B5 model. The final hybrid TDA-DL model achieves state-of-the-art accuracy of 97.4% and an AUC of 0.995 on a holdout test of 395 skin lesions for BCC diagnosis. This study demonstrates that telangiectasia features improve BCC diagnosis, and TDA techniques hold the potential to improve DL performance.

## Introduction

Over two million cases of basal cell carcinoma (BCC) are diagnosed yearly in the USA [1]. The initial diagnosis of BCC includes a visual inspection by a dermatologist or a mid-level practitioner (nurse practitioner or physician assistant), often with a dermatoscope. If the diagnosis is unclear, or if confirmation is needed, an invasive procedure such as a biopsy is performed. Recent research has aimed to improve diagnostic accuracy and minimize the number of biopsies through automatic image processing. In some cases, deep learning (DL) methods applied in dermoscopy have outperformed dermatologists [2–5]. Skin cancer diagnosis from digital images has advanced by implementing DL and, in some cases, fusion ensembles employing DL, metadata, and handcrafted features [6–13].

Telangiectasia or narrow blood vessels within the skin lesions are a critical clinical indicator of BCC. Studies have detected these blood vessels through handcrafted pixel- or patch-based techniques [14, 15]. Cheng et al. [14] investigated a local pixel color drop technique to identify vessel pixels. Kharazmi et al. [15] applied independent component analysis, k-means clustering, and shape for detecting vessels and other vascular structures. Kharazmi et al. [16] detected vessel patches by using a stacked sparse autoencoder (SSAE) as their DL model. Maurya et al. [17] employed DL to segment these vessels semantically, a dermoscopic-feature-driven approach also used by Nambisan et al. to detect dots and globules [18].

Cheng et al. [19] used an adaptive critic design approach to detect and use these vessels for BCC classification. Kharazmi et al. [15] used a random forest–based classifier to diagnose BCC with color and texture features. Kharazmi et al. [20] used a combination of SSAE and patient metadata for BCC diagnosis. Serrano et al. [21] used clustering-based color features and GLCM-based texture features to train VGG16 and MLP models for DL-based BCC classification. All these studies lack the utilization of deep learning–based vessel segmentation and classification together to achieve a BCC diagnosis. This study aims to close this gap by deploying a specific statistical analysis, i.e., Topological Data Analysis, in conjunction with deep learning–based segmentation of telangiectasia, to ultimately perform a BCC classification from digital images.

Topology is a branch of mathematics concerned with the properties of geometric objects that are preserved when the object is stretched, bent, or otherwise deformed. Topological Data Analysis (TDA) is an area of mathematics and data analysis that uses tools from topology to study the shape of data. It is a relatively newer research field that is now increasingly used for image classification, feature extraction, and image analysis [22–27]. The main idea behind TDA is that the shape of the “point cloud” or clusters of data points can reveal important data properties data that may not be immediately apparent from other types of analysis. For example, TDA can be used to identify clusters or groups of data points, detect patterns or trends in the data, and extract features or characteristics that persist along multiple higher dimensional scales. Hu et al. [25] used TDA-based methods for skin lesion segmentation and classification. Bendich et al. [26] employed TDA-based persistence diagrams to find metadata correlations to the brain artery trees, establishing a correlation between age and brain artery tree topology.

This study explores TDA’s ability to extract features from telangiectasia and color-spaces to improve EfficientNet-B5 pre-trained model performance.

This study makes the following unique contributions to the existing literature on automatic BCC diagnosis:

- Integrating a clinically observable physical feature: telangiectasia, with a DL-TDA model to improve diagnosis based on digital medical images
- Demonstrating an alternative, less computationally intensive TDA model for medical image diagnosis.

## Materials and Methods

### Image Datasets

This study uses BCC and benign dermoscopic skin cancer images derived from 3 datasets: the HAM10000 dataset (ISIC 2018) of Tschandl et al. [27], a publicly available skin lesion dermoscopy dataset containing over 10,000 skin images for seven diagnostic categories, the ISIC 2019 dataset [27–29], and datasets R43 from NIH studies CA153927-01 and CA101639-02A2 [30]. The U-Net model is trained on 1000 BCC images, 127 of which come from the HAM10000 dataset, 90 from ISIC 2019, and 783 from the NIH study dataset. We use 1000 non-BCC images from the HAM10000 dataset for our DL BCC diagnostic model. The 1000 non-BCC lesions, along with their distribution in the dataset, are:

- Benign Keratosis: 400
- Nevus: 400
- Actinic Keratosis: 67
- Dermatofibroma: 67
- Vascular Lesion: 66

These five non-BCC conditions listed, overall, have fewer telangiectasia than BCC, and if they have these vascular structures, they are different than BCC telangiectasia. Two of the most common telangiectasia in these non-BCC conditions—hairpin vessels in benign keratosis and comma vessels in nevus—differ from BCC telangiectasia, which have a different architecture. The BCC telangiectasia, in contrast, are often of one of two types: long, thin, and wavy, so-called serpiginous telangiectasia, or trunk and branches connected vessels with varying diameter, so-called arborizing telangiectasia [19]. Clinically, the majority of four of these non-BCC structures, all except vascular lesions, lacked vascular structures, a more inclusive definition of vessels than telangiectasia, as used by [15]. All vascular lesions had vascular structures.

**Fig. 1:**
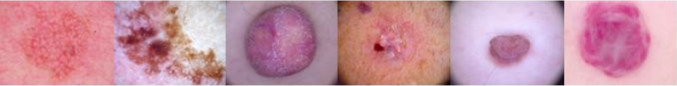
From left: actinic keratosis, benign keratosis, dermatofibroma, basal cell carcinoma, nevus, and vascular lesion

The 1000 BCC images in the dataset are the same as the U-Net model. All the images are 8-bit RGB of size 450 × 600 from the HAM10000 dataset and 1024 × 768 from the NIH study dataset. Example images of these skin lesions are shown in Fig. 1.

### Pre-Processing

**Fig. 2:**
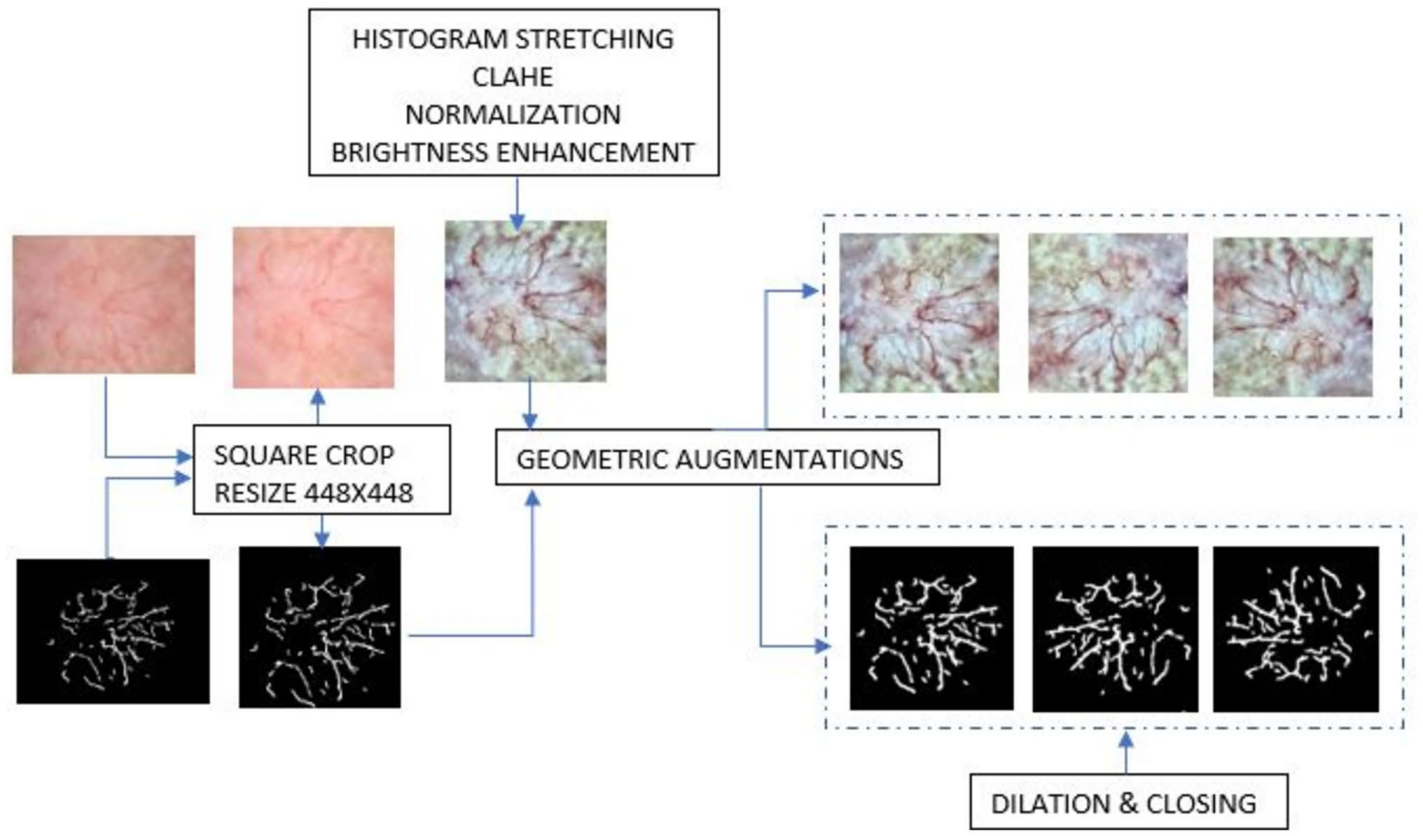
Pre-processing flowchart

Since the skin lesion images are from different datasets and include different resolutions, all the images are square cropped, centering on the lesion area, and resized to 448 × 448. The size 448 × 448 was chosen as it is closest to the smallest size of the skin lesions in the dataset. For the U-Net model, there is an extra step where the images are processed with histogram stretching, contrast limited adaptive histogram equalization (CLAHE), normalization, and brightness enhancement (to make vessels brighter and distinguishable) [17]. The ground truth vessel masks are dilated with a 3 × 3 structuring element and closed with a 2 × 2 structuring element. We perform geometric augmentations: rotation of + 30° to − 30° in reflect mode (to preserve vessel continuity), horizontal and vertical flip, width shift with a range of (− 0.2, + 0.2), height shift with a range of (− 0.2, + 0.2), and shear with a range (− 0.2, + 0.2). Figure 2 shows the steps of pre-processing.

### Proposed Methodology 

**Fig. 3:**
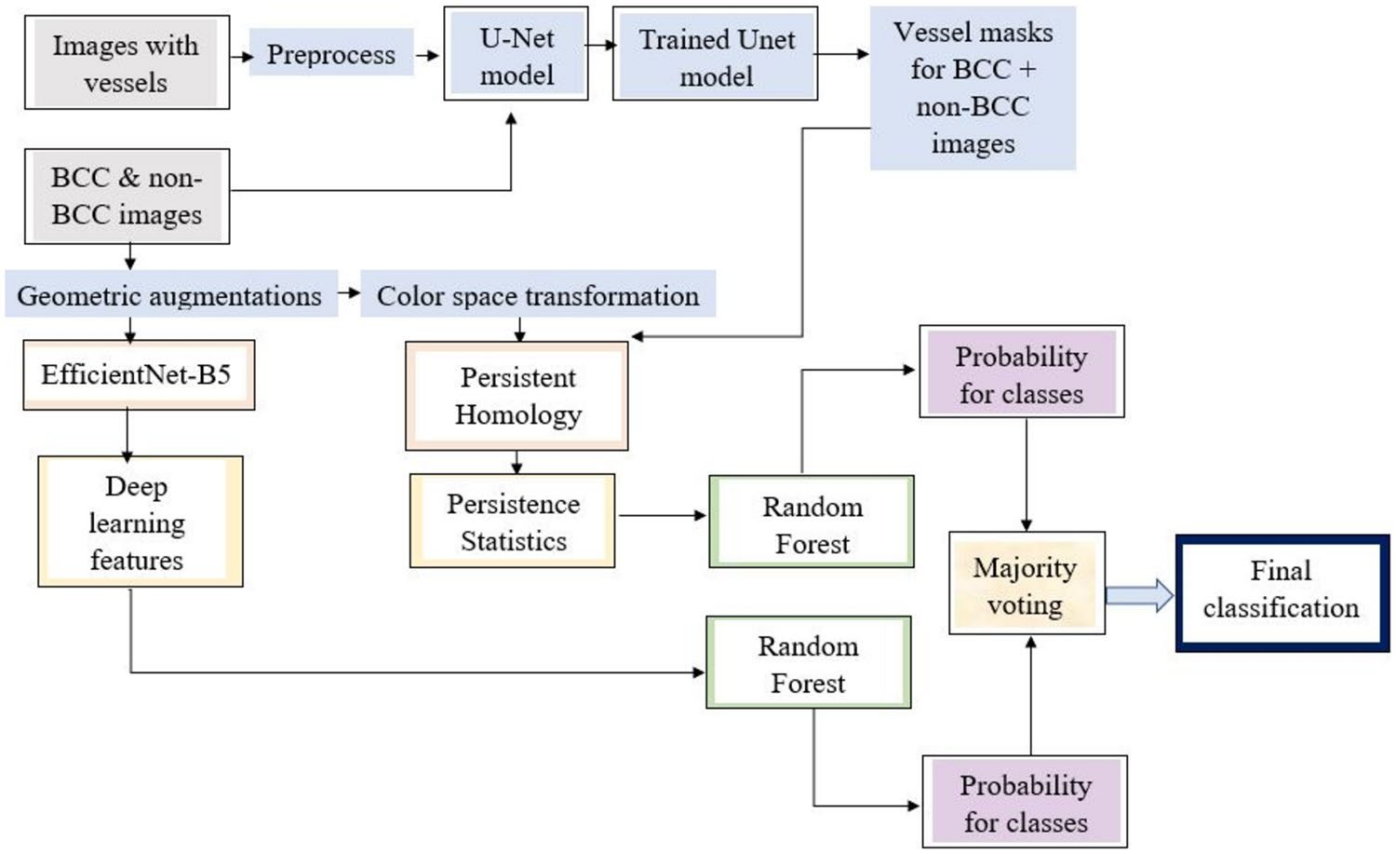
Pipeline investigated employing a hybrid TDA-DL method for BCC classification

Figure 3 presents our five-component hybrid TDA and DL model pipeline investigated in this study, including:

- Part 1: U-Net model that semantically segments telangiectasia in both BCC and non-BCC skin lesion images
- Part 2: TDA framework that generates Persistence Statistics from telangiectasia and color spaces of the images
- Part 3: DL classification model based on EfficientNet-B5 for feature extraction from skin lesions
- Part 4: Random Forest classifiers that generate class probabilities from DL and TDA features
- Part 5: Voting between the probabilities generated in Part 4 to yield a final BCC vs non-BCC classification

#### Part 1: U-Net Model for Segmenting Telangiectasia

U-Net-based segmentation models are widely used in medical image segmentation [31]. The U-Net model and its hyperparameters are taken from [17] for this study. The model produces binary vessel masks for BCC and non-BCC lesion images. A TDA framework uses these binary vessel masks (as explained in detail in the subsequent sections) to generate topological features.

#### Part 2: Topological Data Analysis (TDA), Persistent Homology and Persistence Statistics

**Fig. 4:**
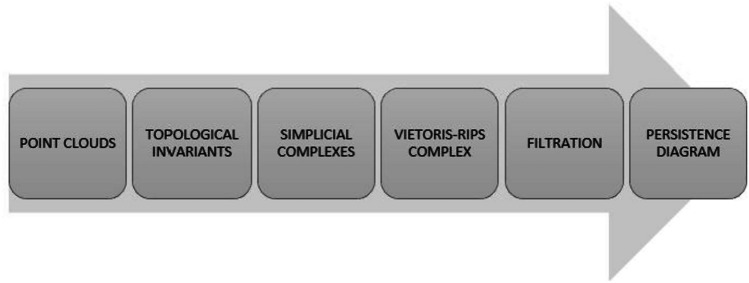
Flow of the persistent homology algorithm to generate persistence diagrams

Topological Data Analysis (TDA) applies the concepts and methods of topology for the analysis and visualization of complex data. Persistent homology (PH), a statistical tool of TDA, can detect topological features of the data that persist over larger scales and long intervals of time. PH accounts for the topological features, i.e., connected components in dimension 0, loops in dimension 1, and voids in dimension 2 by creating persistence diagrams [22–26]. The persistent homology algorithm follows the steps shown in Fig. 4.

The main steps of the process are described below:

(A) Point clouds: Point clouds are collections of data points that reflect the geometry and spatial relationships of a real-world object or environment in a high-dimensional space, most often a three-dimensional (3D) space. The x, y, and z coordinates of each point in a point cloud, as well as any other qualities like color or intensity, are used to identify each point’s location in the space. Point clouds serve as the pixel intensity values in this study.
(B) Topological invariants: Topological invariants are topological space–related mathematical numbers or qualities that are true even if the space is altered in some way. These invariants offer a mechanism to categorize and separate various topological spaces according to their inherent characteristics. Betti numbers are also a type of topological invariant representing the total number of holes in a space of various sizes. Higher Betti numbers count higher-dimensional holes. The first Betti number counts independent loops, while the zeroth Betti number counts connected components. Topological invariants are frequently generated from algebraic structures known as homology groups or cohomology groups in the setting of algebraic topology, which examines the algebraic features of topological spaces. Chains or cochains, formal combinations of simplices or cells in a topological space, are used to create these groups.
(C) Simplicial complex: In the study of combinatorial topology and geometry, a simplex is a fundamental geometric object. It is an extension of the 2-dimensional simplex idea of a triangle to higher dimensions. The convex hull of (*n* + 1) affinely independent points in Euclidean space is formally referred to as an *n*-dimensional simplex. A simplex is the “simplest” conceivable polytope in *n*-dimensional space; equivalently, it is a geometric object [22–26]. Here are a few instances:

- A vertex of a zero-dimensional simplex is represented by a single point
- A line segment joining two points is referred to as a one-dimensional simplex
- A triangle having three vertices and three edges is referred to as a two-dimensional simplex
- A tetrahedron with four vertices, six edges, and four triangular faces is referred to as a three-dimensional simplex

Topological invariants can be computed from the simplicial complex by counting the number of simplexes of different dimensions that make up the complex. Let *V* be a set of vertices. A subset *S* of *V* is called a simplex of dimension *n* if it contains *n* + 1 elements that are affinely independent, meaning that the points do not lie in a lower-dimensional hyperplane. The elements of *S* are called the vertices of the simplex. A simplicial complex *K* is a collection of simplexes in *V* that satisfies the following conditions [22–26]:

- Any face of a simplex in *K* is also in *K*, meaning that if *S* is a simplex in *K*, then every subset of *S* that is a simplex is also in *K*.
- The intersection of any two simplexes in *K* is either empty or a face of both.

**Fig. 5:**
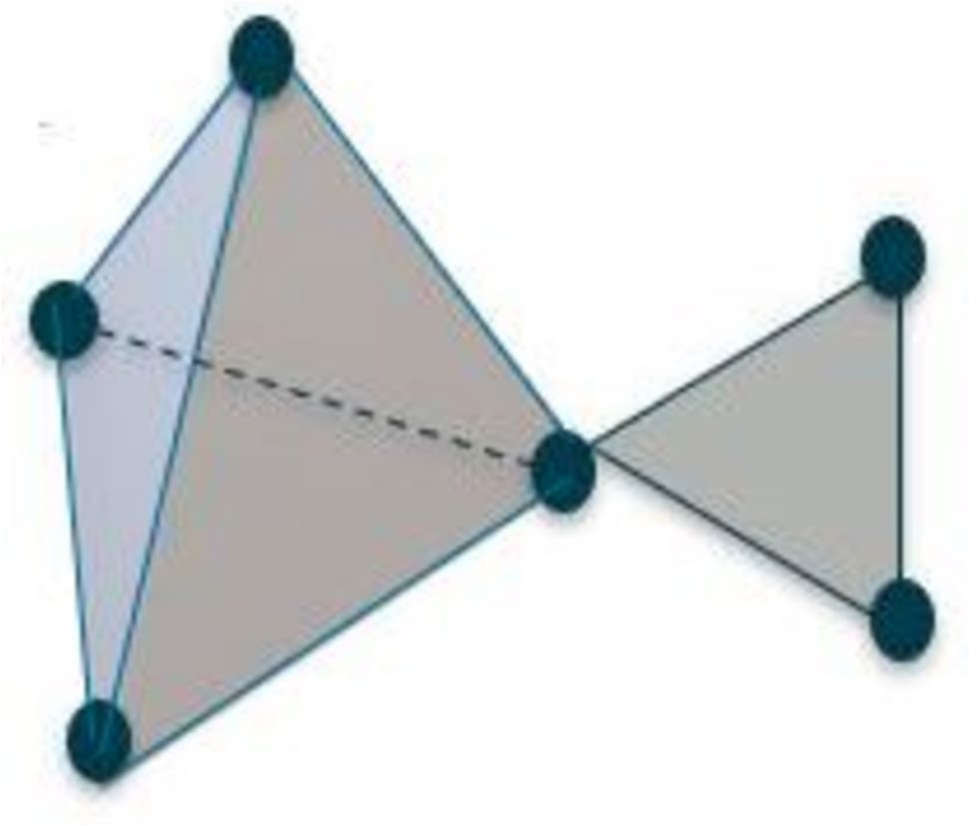
Simplicial complex containing a 3D simplex (tetrahedron) and a 2D simplex (triangle)

Figure 5 shows a simplicial complex that includes a tetrahedron and a triangle.

(D) Vietoris-Rips complex: This study utilizes the Vietoris-Rips complex to produce simplicial complexes from the image datasets. It is built by joining together spatial pairs of points that are relatively close to one another and then joining together higher-dimensional simplexes while considering the connectivity of the lower-dimensional simplexes.

Formally, for a set of points *P* of dimension *d*, where *P* is a subset of Rd, and then the Vietoris-Rips (VR) complex *V*ϵ(*P*) at scale ϵ (the VR complex over the point cloud *P* with parameter ϵ) is defined as [22–26]:

$${V}{\in }\left(P\right)=\left\{\sigma \subseteq P|d\left(u,v\right)\le \epsilon , \forall u\ne v\in \sigma \right\}V$$

Hence, for a set of data points in *P*, a simplex σ (a subset of *P*) is included, if the points in σ are all within a distance of ϵ from each other. As a result, a collection of subsets of *P* is obtained, that are all simplices, or a simplicial complex of *P*.

(E) Filtration: By varying the values of ϵ to different levels, it can be discovered what appears to produce a significant VR complex. If ϵ is set too small, the complex might just include the initial point cloud or a sparse number of edges connecting the points. On the other hand, the point cloud will merge into one enormous ultradimensional simplex if ϵ is set too large. In order to truly find patterns in a simplicial complex, the parameter ϵ must be repeatedly changed (and generate new complexes) from 0 to a maximum that yields a single huge simplex. Then, the persistence diagrams illustrate which topological features are created and destroyed as ϵ keeps rising. It is assumed that the features which persist over a long period of time are significant and vice versa. This process is called filtration.
(F) Persistence diagrams: A persistence diagram is a graphical representation of this process, which consists of a collection of points in a two-dimensional plane. Each point in the diagram represents a topological feature and its corresponding lifespan or persistence, defined as the difference between the scale at which the feature was born and the scale at which it died out. The diagram’s horizontal axis represents the birth values of the topological features, while the vertical axis represents their death values. The diagonal line in the diagram represents features with the same birth and death values and is called the diagonal or the “line of equality.”

Zero-dimensional persistent homology and 1D persistent homology refer to the analysis of topological features in different dimensions using the persistent homology framework. 0D persistent homology analyzes connected components or clusters in a data set. It captures the evolution of these connected components as a parameter, typically related to distance or scale, varies. By systematically increasing or decreasing the parameter, 0D persistent homology tracks the birth and death of connected components. In 0D persistent homology, the filtration complex is constructed by associating each data point in the set with a 0-dimensional simplex. Initially, each data point is a separate connected component. As the parameter increases or decreases, connected components may merge or disappear, resulting in changes in the topology of the data set. The persistence intervals, or barcode intervals, represent the lifespan of the connected components, indicating when they are born and when they die.

One-dimensional persistent homology focuses on analyzing loops or cycles in a data set. It captures the evolution of these loops as the filtration parameter varies. By systematically changing the parameter, 1D persistent homology tracks the birth and death of loops. In 1D persistent homology, the filtration complex is constructed by considering both the data points and the edges connecting them. Initially, each data point is a 0-dimensional simplex, and each edge is a 1-dimensional simplex. As the parameter increases or decreases, edges may form loops or cycles, merge with existing loops, or disappear. The persistence intervals represent the lifespan of the loops, indicating when they are born and when they die.

From this point onwards, the persistence diagrams corresponding to 0D and 1D persistent homology are referred to as P0 and P1.

**Fig. 6:**
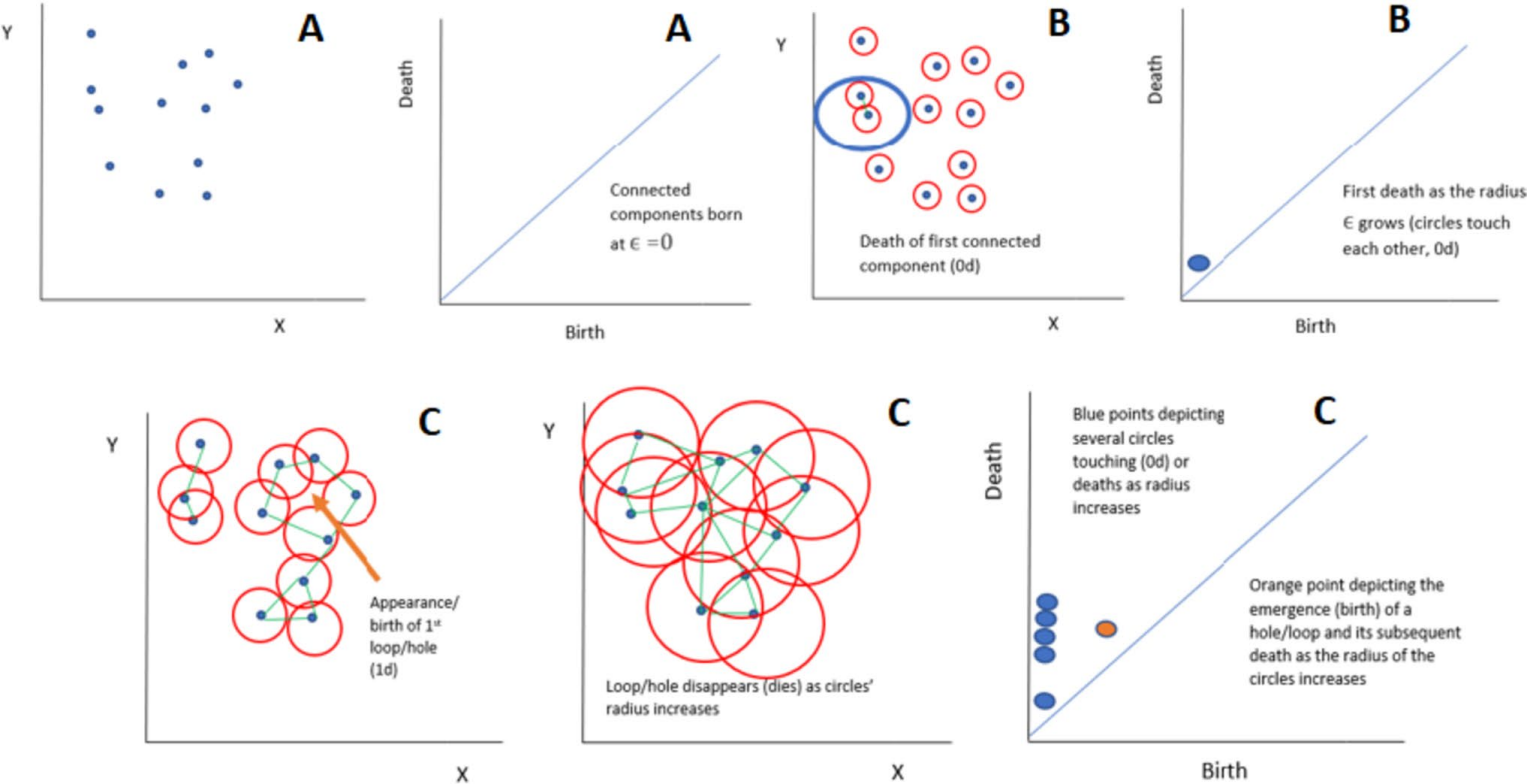
Persistent homology filtration process leading to formation of birth–death pairs in the persistence diagram

One approach to understanding this filtration process involves creating a sequence of growing spheres centered on each point and connecting those with overlapping spheres with edges or triangles. Figure 6 illustrates this process.

(A) The process starts with a collection of data points (point clouds) in 2D space. At this point, the value of ϵ or the radius of the spheres is 0. Hence the connected components are born at *x* = 0. Since there has been no death or “overlap,” there is no corresponding *y* value.
(B) As the concentric spheres around the datapoints increase in size/radii (ϵ increases), the first connected components die or overlap, giving us the first death. Hence, the first birth–death pair point on the corresponding persistence diagram is observed with birth at *x* = 0 and death at *y* > 0, where *x* and *y* both correspond to the radius ϵ of the spheres.
(C) At this stage, with the radius or ϵ increasing, more deaths or overlaps happen, leading to more deaths and larger values of *y*, but there is also the emergence of a loop, hence a birth value for 1D homology. This loop finally disappears in the second substage. Hence, a birth and death value for *x* and *y* is created, both greater than zero and accounted for by the orange point in the corresponding persistence diagram.

Therefore, the persistence diagram provides a global summary of the topological features of a dataset, capturing both their presence and persistence over different scales.

**Fig. 7:**
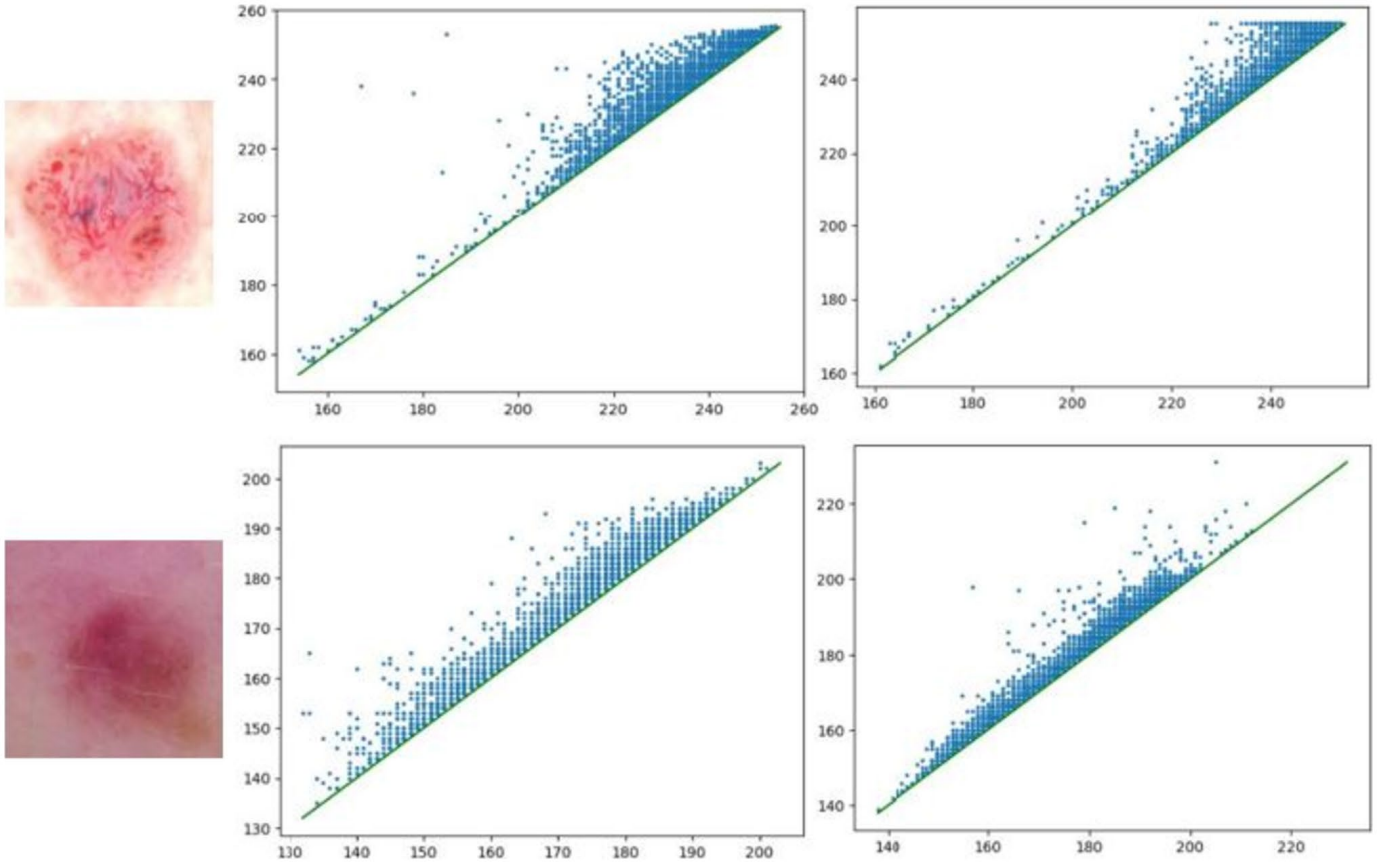
Top row, from left: BCC skin lesion; its corresponding P0 persistence diagram and P1 persistence diagram for the red plane; bottom row, from left: non-BCC (actinic keratosis) skin lesion; its corresponding P0 persistence diagram and P1 persistence diagram for the red plane

For image classification, one channel is used at a time from a 3-channel color space, for example, red color plane from the RGB color space, grayscale, or binary image. PH is used for image analysis by treating image pixels as point clouds, where point clouds are a collection of data points in a high-dimensional space. The shape of the point cloud can reveal important data properties and that can be used to identify patterns in images, such as textures or shapes, and to measure the similarity between different images. Figure 7 shows persistence diagrams P0 and P1 for a BCC and non-BCC image for the red color channel from the RGB color space. It is noticeable, even by visual observation, that the birth–death pairs for both images seem distinguishable.

##### 2.3.2.1 Persistence Statistics for Telangiectasia

In the context of a digital image, a point cloud is a set of points in a high-dimensional space representing each pixel’s position and color information. Each point in the point cloud corresponds to a single pixel in the image, and its position in the space is determined by its *x* and *y* coordinates, while additional dimensions or attributes can represent its color. For the dataset in this study, each channel of a 3-channel image (example RGB) is treated as a grayscale image with pixel intensity values ranging from 0 to 255 [25]. This forms the initial point cloud for the subsequent persistent homology process.

There are a total of six different sets of persistent homology features called “persistence statistics” (PS) calculated in this study. 5 channels are extracted from 3 different color spaces, namely, R, G, and B from the RGB color space, V from the HSV color space, and Z from the XYZ color space. The 6th set of persistence statistics is calculated from the predicted telangiectasia masks. For all the channels, both *P*0 and *P*1 are generated, leading to a total of 32 PS features per set. Hence, the total number of features generated using PS is 32 × 6 = 192.

**Fig. 8:**
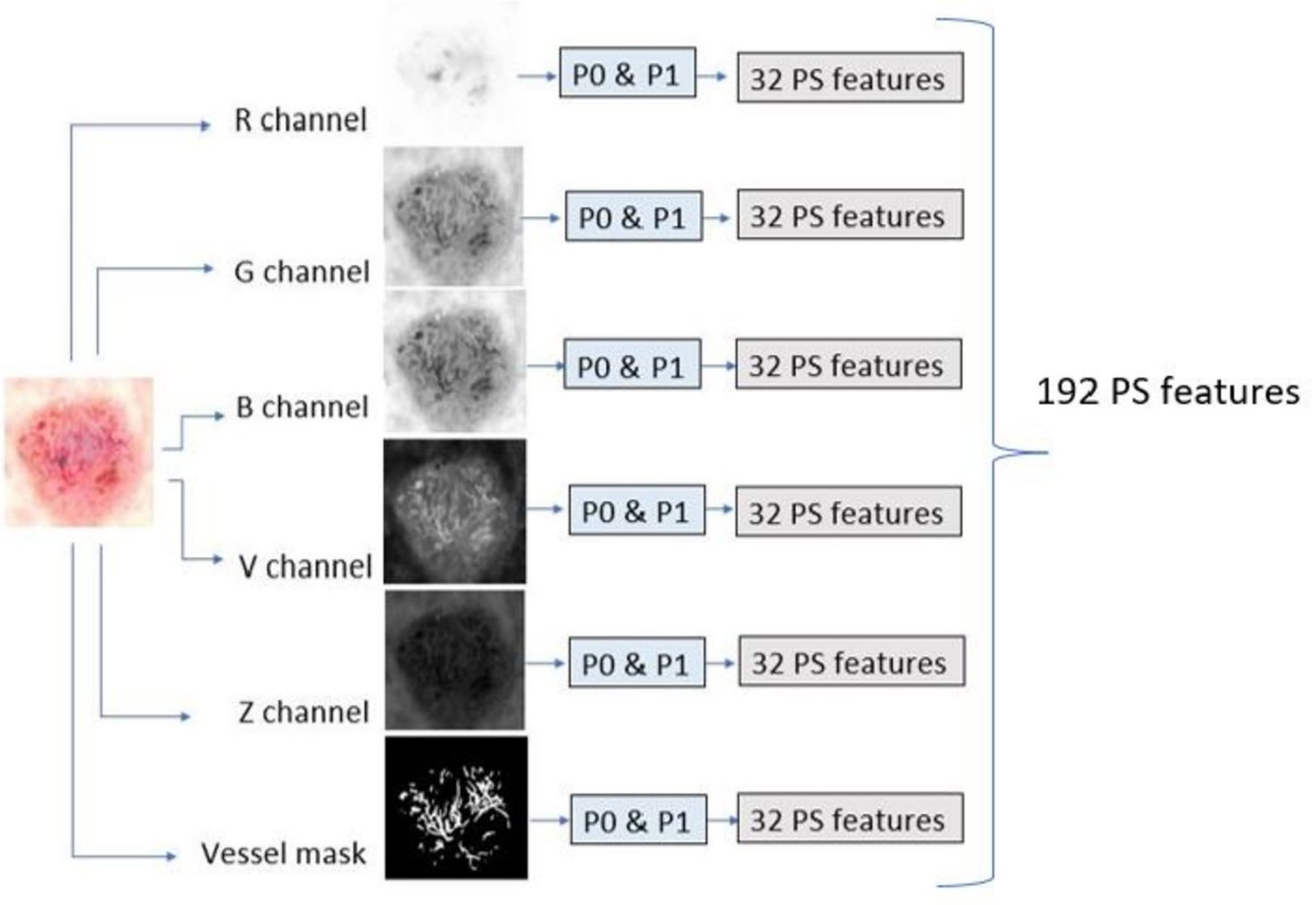
Generation of persistence statistics–based features

Figure 8 illustrates the PS features. As shown in Fig. 7, the persistence diagram *P* contains collections of pairs of points that represent the birth and death values of topological features. Our persistence statistics include three quantities that summarize this information in persistent diagrams: total persistence, mid-life coordinates, and normalized lifespan [25]. If birth is denoted by *b* and death is denoted by *d*, *d* − *b* is the lifespan of the topological feature. It represents the length of time that the corresponding feature persisted during the filtration process. Total persistence is then defined as the sum of the persistence values over all points in the diagram. Mathematically, this can be expressed as:

$${\mathrm{L}}_{\mathrm{i}}={\Sigma }_{(b,d)\in {P}_{i}}d-b$$

where *i* = 0,1 corresponding to *P*0 and *P*1. Total persistence provides a global measure of the complexity or richness of a dataset’s topological structure by considering all the topological features and their persistence over different scales. Another statistic is midlife coordinates, expressed mathematically as:

$${\mathrm{M}}_{\mathrm{i}}=(\mathrm{b}+\mathrm{d})/2$$

The third measure is normalized lifespan. It measures the relative persistence or robustness of topological features in a dataset, considering their lifespans and the overall complexity of the persistence diagram. We calculate the normalized lifespan *p*_i_ for each point in the diagram as its persistence divided by the total persistence:

$${\mathrm{p}}_{\mathrm{i}}=(\mathrm{d} - \mathrm{b})/\mathrm{Li}$$

The normalized lifespan *p*_*i*_ is a measure of the relative persistence or robustness of a topological feature in comparison to the overall complexity of the persistence diagram [23]. It indicates the proportion of the total persistence contributed by the corresponding feature and provides insight into how long-lasting and persistent the feature is. *M*_*i*_ and *p*_*i*_ are empirical distributions [25], and we apply standard statistical measurements on these distributions to calculate our feature vector. Table 1 shows the 32 topological features we calculated for each image in our dataset.

**Table 1:** Persistence statistics calculated for our methodology

**Feature number**	**Feature name (i = 0,1)**	**Description**
1 to 4	Pi_mean_midlife Pi_mean_normalized_lifespan	Means of *M*_*i*_ and *p*_*i*_
5 to 8	Pi_std_midlife Pi_std_normalized_lifespan,	Standard deviation of *M*_*i*_ and *p*_*i*_
9 to 12	Pi_skew_midlife Pi_skew_normalized_lifespan	Skewness of *M*_*i*_ and *p*_*i*_
13 to 16	Pi_kurtosis_midlife Pi_kurtosis_normalized_lifespan	Kurtosis of *M*_*i*_ and *p*_*i*_
17 to 20	Pi_median_midlife Pi_median_normalized_lifespan	Medians of *M*_*i*_ and *p*_*i*_
21 to 24	Pi_perc25_midlife Pi_perc25_normalized_lifespan	25th percentile of *M*_*i*_ and *p*_*i*_
25 to 28	Pi_perc75_midlife Pi_perc75_normalized_lifespan	75th percentile of *M*_*i*_ and *p*_*i*_
29 to 32	Pi_interquart_midlife Pi_interquart_normalized_lifespan	Interquartile ranges of *M*_*i*_ and *p*_*i*_

#### Part 3: EfficientNet-B5 for Feature Extraction from Skin Lesions

A family of convolutional neural network (CNN) models called EfficientNet has attained cutting-edge performance on various computer vision applications while retaining a manageable number of parameters [32–34]. By properly scaling the network in several dimensions, EfficientNet’s major goal is to address the trade-off between model size and accuracy. In the past, scaling a model meant individually expanding its depth, width, or resolution. EfficientNet, on the other hand, suggests a compound scaling technique that considers depth, width, and resolution all at once. The compound scaling technique also ensures that the model can be efficiently fine-tuned on smaller datasets without overfitting. Lama et al. [35, 36] successfully employed EfficientNet-based DL models for lesion segmentation and hair detection. Hence, for this study, an EfficientNet-based model is chosen, specifically EfficientNet-B5, for extracting deep-learning features for the classification model.

The top layers from the original EfficientNet-B5 model are removed as it was designed for 10-class classification instead of binary classification. They are replaced with a global average pooling layer, a dropout layer, and a final dense layer. The initial input image size for the model is 448 × 448 × 3. It has 14 phases and is first trained for classification, then feature extraction using the trained model. It begins with a 3 × 3 filter convolution, batch normalization, and swish activation function for the classification stage, cutting the image dimensions in half from 448 to 224 and raising the number of channels from 3 to 48. As a result, the feature map’s measurements are 224 × 224 × 48. Stage 2 is composed of three layers of an MBConv1 block with a 3 × 3 filter, which reduces the number of channels while maintaining the resolution of stage 1 to produce a feature map with dimensions of 224 × 224 × 24. Stages 3 (five layers), 4 (five layers), and 5 (seven layers) employ three MBConv6 blocks, each with a kernel size of 5 × 5, to gradually decrease the resolution while expanding the size of the feature map to 28 × 28 × 128 (the stage’s finish). Stages 6 (7 layers), 7 (9 layers), and 8 (3 layers) each apply three more MBConv6 blocks with kernel sizes of 3 × 3, 5 × 5, and 3 × 3 to create a feature map with a final dimension of 14 × 14 × 512. A feature map with the dimensions 14 × 14 × 2048 is produced at stage 9 using a 1 × 1 convolution with 2048 filters. Stages 10 and 11 maintain the feature size from the preceding layer while applying batch normalization and Softmax activation. Stage 12 uses global average pooling to increase the resolution to 2048 followed by stages 13 and 14 leading to the final classification: a dropout and dense layer. After the 200th layer, the model is fine-tuned, and the best model is saved. After the global average pooling layer, at stage 12, feature extraction is carried out, producing a 2048-dimensional feature vector for the training, validation, and test sets.

Table 2 displays the phases, procedures, resolutions, channels, and layers.

**Table 2:** EfficientNet-B5-based deep learning model

**Stage**	**Operator**	**Resolution**	**Channels**	**Layers**
1	Conv 3 × 3 + BN + Swish	224 × 224	48	1
2	MBConv1, k3 × 3	224 × 224	24	3
3	MBConv6, k5 × 5	112 × 112	40	5
4	MBConv6, k5 × 5	56 × 56	64	5
5	MBConv6, k5 × 5	28 × 28	128	7
6	MBConv6, k3 × 3	28 × 28	176	7
7	MBConv6, k5 × 5	14 × 14	304	9
8	MBConv6, k3 × 3	14 × 14	512	3
9	Conv 1 × 1	14 × 14	2048	1
10	BN	14 × 14	2048	1
11	Activation	14 × 14	2048	1
12	Global Average Pooling	2048	1	1
13	Dropout	2048	1	1
14	Dense	1	1	1

#### Parts 4 and 5: Class Probabilities and Majority Voting

The 2048-dimensional feature vector from the EfficientNet-B5 model and the 192-dimensional TDA-PS feature vector are both used as inputs for two different random forest classifiers. These random forest ensemble learners generate probabilities for each class (BCC and non-BCC), resulting in 4 probability values:

- DL_prob_1: probability of a lesion being BCC based on DL features
- DL_prob_0: probability of a lesion being non-BCC based on DL features
- TDA_prob_1: probability of a lesion being BCC based on TDA features
- TDA_prob_0: probability of a lesion being non-BCC based on TDA features

For each image, the probabilities are compared and the class with the highest probability is chosen as the final class.

### Training Details

Both deep-learning models, U-Net and EfficientNet-B5, were built using Keras with a Tensorflow backend in Python 3.7 and trained using a single 32 GB Nvidia V100 graphics card. Hyperparameters for the U-Net model are the same as for Maurya et al. [17].

The hyperparameters for the EfficientNet-B5 model are listed in Table 3. For the random forest classifier, 1000 estimators are used with the Gini index criterion. The minimum samples per split are 2 with bootstrapping.

**Table 3:** Hyperparameters for the EfiicientNet-B5 model

**Hyperparameter**	**Values**
Fine-tuning layer	200
Epochs	120
Learning rate	0.0001
Batch size	20
Loss function	Binary cross-entropy
Optimizer	Adam
Early stopping criteria	Validation loss
Patience	5
Dropout rate	0.2

## Experimental Results

This section discusses the results of various stages of our study. All results listed were evaluated on the holdout BCC vs non-BCC test set of 395 skin lesion images (195 BCC and 200 no-BCC). The evaluation metrics used are accuracy, sensitivity, specificity, and precision (PPV) [37, 38].

### U-Net Telangiectasia Segmentation Results

**Fig. 9:**
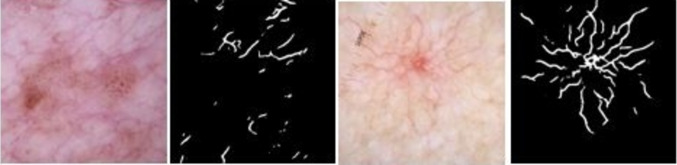
From left: non-BCC image with vessel mask prediction, BCC image with vessel mask prediction

The first set of results come from the U-Net model for segmenting telangiectasia. Figure 9 shows examples of non-BCC and BCC images with their corresponding predicted vessel masks. The automatic telangiectasia masks generated were non-blank for most of the five non-BCC diagnoses. The proportions that were non-blank were benign keratosis 33/400, nevus 24/400, actinic keratosis 9/67, dermatofibroma 3/67, and vascular lesions 3/66. It can be seen that the U-net model can segment vessels in both types of lesions even though the vessels are distinguishable. As seen in the next section, persistence statistics exploit this discriminative feature and improve classification Table 4.

**Table 4:** Random forest classification with PS for R, G, B, V, Z, and vessels

**Model**	**Feature set size**	**Accuracy**	**Sensitivity**	**Specificity**	**Precision**
R, G, B	32 × 3 = 96	0.900	0.875	0.928	0.875
R, G, B, V	32 × 4 = 128	0.916	0.889	0.934	0.889
R, G, B, V, Z	32 × 5 = 160	0.920	0.900	0.945	0.900
R, G, B, V, Z, vessels	**32** × **6 = 192**	**0.943**	**0.938**	**0.950**	**0.950**

The bold values highlight the best classification results for the different metrics examined

### BCC Classification with Persistence Statistics and Random Forest Classifier

Following the telangiectasia segmentation, the next set of results come from persistence statistics calculated from the predicted binary telangiectasia masks, as well as the 5 color channels R, G, B, V, and Z. Table 5 shows the BCC vs non-BCC classification results of a random forest classifier trained on the PS features. There is a 2.3% jump in accuracy, 3.8% jump in sensitivity, 0.5% jump in specificity, and 5% jump in precision after adding vessel features, signaling the importance of telangiectasia in improving BCC diagnosis.

**Table 5:** Metric improvements with subsets of PS features

**Feature set size**	**Accuracy**	**Sensitivity**	**Specificity**	**Precision**
First 50	0.867	0.855	0.866	0.867
First 90	0.885	0.867	0.889	0.883
First 130	0.902	0.890	0.913	0.900
First 170	0.911	0.905	0.925	0.910
All 192	**0.943**	**0.938**	**0.950**	**0.950**

The bold values highlight the best classification results for the different metrics examined

To ensure features were not redundant, a feature importance test was also performed with random forest. Subsets of the 192 features were chosen and all the metrics shown in Table 5 were recalculated. Table 6 shows that metrics improve considerably after continuously adding PS features. It was observed that all 192 features are needed for high diagnostic accuracy.

**Table 6:** Performance comparison of different deep learning models

**Model**	**Feature set size**	**Accuracy**	**Sensitivity**	**Specificity**	**Precision**
InceptionV3-FT	2048	0.920	0.910	0.942	0.934
EfficientNet-B0-FT	1280	0.936	0.925	0.947	0.920
EfficientNet-B5-FT	**2048**	**0.959**	**0.942**	**0.950**	**0.950**

The bold values highlight the best classification results for the different metrics examined

### BCC Classification with EfficientNet-B5 and Feature Vector Generation

**Fig. 10:**
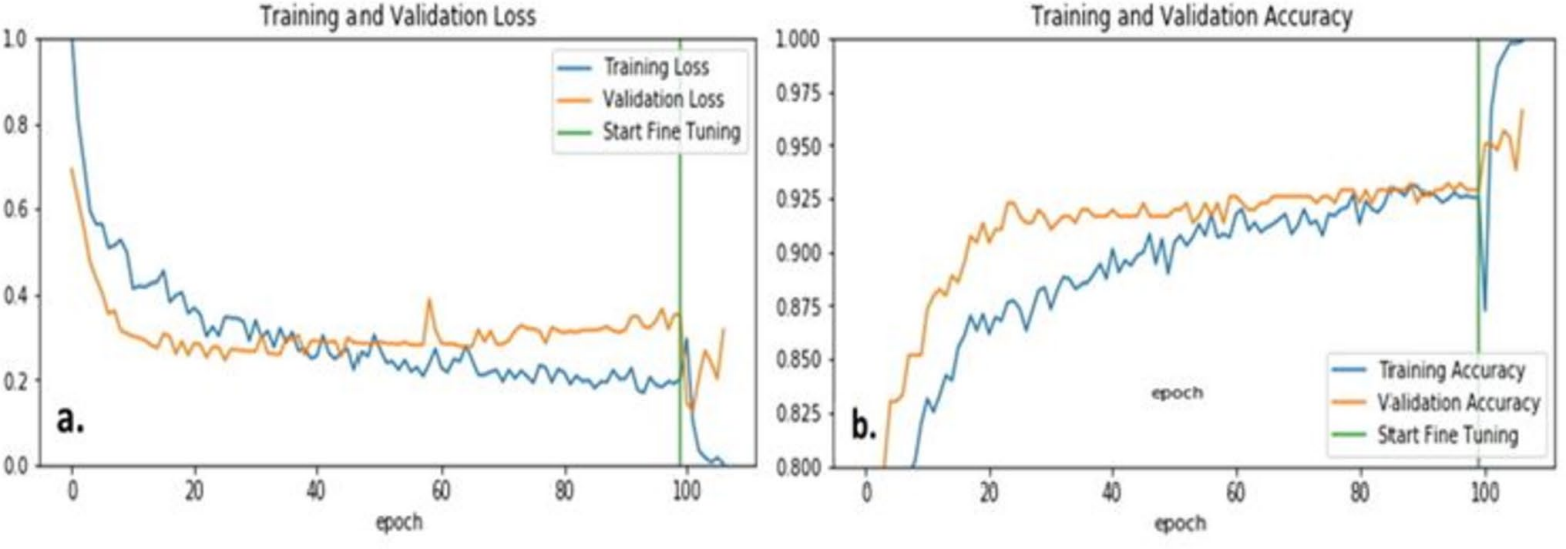
Training and validation accuracy and loss curves for fine-tuning EfficientNet-B5on BCC vs non-BCC dataset

The next set of results is generated from fine tuning three pre-trained DL models on the BCC vs non-BCC dataset. The best results were achieved with the EfiicientNet-B5 model, and hence, it was chosen for feature extraction after fine-tuning. Tables 5 and 7 show that the TDA-based random forest model performs better than EfficientNet-B0 and InceptionNetV3 but slightly worse than EfficientNet-B5. Figure 10 shows the loss and accuracy plots for the EfficientNet-B5 model before and after fine-tuning. The 2028 feature vector is generated at this stage.

**Table 7:** Performance comparison of DL model, PS model, and hybrid TDA-DL model

**Model**	**Feature set size**	**Accuracy**	**Sensitivity**	**Specificity**	**Precision**
DL-EfficientNet-B5	2048	0.959	0.942	0.950	0.950
TDA (PS based)	192	0.943	0.938	0.950	0.950
DL-TDA Hybrid without vessels	2208	0.965	0.963	0.970	0.971
DL-TDA Hybrid with vessels	2240	**0.974**	**0.972**	**0.978**	**0.979**

The bold values highlight the best classification results for the different metrics examined

### Final BCC vs Non-BCC Classification with Hybrid TDA-DL Model

The final set of results comes from the hybrid TDA-DL model. In this model, voting is done on classification probabilities generated from a random forest classifier trained on DL and TDA features. All the metrics are recalculated as shown in Table 8. The first row shows DL-only classification results, whereas the 2nd row shows TDA-only classification results. Next, adding the TDA-based persistence statistics (PS) features to the 2048 feature vector from EfficientNet-B5 improves the deep-learning results in two folds. First, in the 3rd row, only the color channel–based PS features are used in combination with DL features, and it can be seen that these color PS features improve the DL results, as accuracy is increased by 0.6%, sensitivity by 2.1%, specificity by 2%, and precision by 2.1%. Second, in the fourth row, PS features from vessels are now included, i.e., the full PS feature set (192 features) is combined with the DL set, the hybrid model’s accuracy rises by 1.5%, sensitivity jumps by 3%, specificity jumps by 2.8%, and precision jumps by 2.9%.

**Table 8:** Performance comparison with other studies

**Manuscript**	**Dataset**	**Feature categories**	**Accuracy**	**Sensitivity**	**Precision**
Kharazmi et al. 2017	659; 299 BCC and 360 non-BCC	Vascular features	0.965	0.904	0.952
Kharazmi et al. 2018	1199; 599 BCC and 600 non-BCC	Patient profile information & SAE feature learning	0.911	0.853	0.877
Serrano et al. 2022	692 BCC and 671 non-BCC	Color and texture features	0.970	**0.993**	0.953
DL-TDA hybrid	2000; 1000 BCC and 1000 non-BCC	EfficientNet-B5 & localized vessel and global TDA features	**0.977**	0.977	**0.979**

The bold values highlight the best classification results for the different metrics examined

### Performance Comparison with Existing Methods

Table 8 compares the performance of the TDA-DL hybrid model with other published studies on the automation of BCC diagnosis [15, 20, 21]. Kharazmi et al. [15] used vascular features from vessels whereas in [20], they used patient meta-data along with DL-based auto-encoder features. Serrano et al. [21] used annotated features to account for the presence of several clinical biomarkers. The hybrid model in this study achieves higher accuracy and precision overall and produces segmentation telangiectasia as a sub-step. This is the only study (to the best of our knowledge) exploring TDA approaches and focusing on diagnostic improvements attributed to clinical features.

## Discussion

The inclusion of biomarker-driven features for automation of cancer diagnosis is a rapidly growing field. Since telangiectasia is a critical indicator of BCC, the automation of telangiectasia detection is an important step in BCC diagnosis. Studies on this task include ones based on traditional rule–based image processing techniques such as color drop vessel detection [14] and independent component analysis of melanin and hemoglobin components, followed by thresholding and clustering [20]. Deep learning [17] performed this task at a pixel level by a U-Net segmentation model which obtains a Jaccard score within the variation of human observers. The hybrid TDA-DL methodology discussed in this paper is the only study that explores the effects of these precisely segmented telangiectasia on BCC classification.

Just as DL is able to extract abstract features from digital images, TDA has interested researchers because of its ability to extract topological or geometrical properties from data. It has been used extensively and successfully in many applications in medical image analysis [25], biology [39], and neurology [26]. TDA can be applied to data with limited or noisy information since it can work with incomplete or partial data as it captures the multiscale structure of the data. In [25], TDA was used for lesion segmentation and generation of lesion features whereas in [39], persistence homology was used for studying osmolytes molecular aggregation and their hydrogen-bonding network from a local topological perspective. Bendich et al. [26] implemented persistence homology to correlate brain artery trees with the age of their subjects. Brain artery trees are somewhat similar to telangiectasia in their structure. This study applies persistence homology to generate telangiectasia features (persistence statistics) and combines them with DL features learned from a pretrained EfficientNet-B5 model. The TDA features focus on telangiectasia and skin lesions whereas the DL features focus only on whole skin lesions and the combination of these two together form the hybrid TDA-DL model. There have not been any other studies that have explored this aspect of BCC diagnosis.

Ensemble learning through random forest is applied to the hybrid feature set in this study. The initial random forest classification model based solely on persistence statistics derived from the red, green, blue, V channel of HSV color space, and Z channel of XYZ color space cannot outperform deep-learning models. However, after adding persistence statistics (PS) features derived from telangiectasia masks, it was observed that the random forest classifier outperforms Inception-V3 and EfficientNet-B0 models, indicating the importance of this clinical feature in diagnosis. This observation is also significant as deep learning models learn the abstract data with the help of ground truth labels provided to them, whereas TDA-based methods perform feature extraction without ground truth labels, i.e., unsupervised learning. The PS-based TDA model accuracy result is slightly lower than that of the EfficientNet-B5.

TDA features can predict the BCC class more accurately for some test cases missed by deep learning. In the final hybrid DL-TDA model, majority voting is applied to the DL and TDA probabilities. The accuracy improves by almost 2% on the holdout test set. Another important aspect worth mentioning is that the computational cost of calculating the persistence statistics features is significantly lower than for the deep learning features, i.e., they can be calculated without a high-performance GPU. Handcrafted features have also been used for improving diagnosis with fusion [6–13, 19], but usually, feature calculation is feature- and problem-dependent. With TDA analysis, we can bypass those limitations.

Even with the recent improvements in the automation of skin cancer diagnosis, we acknowledge that raising the sensitivity and specificity of these models is an ongoing challenge. As such, the final sensitivity of this study was slightly lesser than the one in [21]. All the comparable studies discussed, which utilize deep learning models, including this study, do not incorporate tests for statistical significance. Choosing the most suitable hypothesis testing method in the context of deep learning poses a challenge and requires significant time and resources. Deep learning has mainly prioritized predictive accuracy and model generalization over group mean comparisons. Nevertheless, there is ongoing research into identifying suitable statistical significance tests for model selection in machine learning. Furthermore, both our study’s datasets and those in comparable research lack diversity in skin color, which restricts their applicability.

Another limitation for this study is that the ground truth vessel mask marking was supervised by a single dermatologist (WVS). Only one team observer (one of AM, DS, SS, or WVS) annotated each mask. While there is considerable overlap in the datasets used in similar studies, the absence of a common dataset with established reference data complicates the process of creating a reliable benchmark for comparison. Therefore, through this research, the telangiectasia masks and corresponding images are openly shared [40].

## Conclusion

This study proposes a deep learning and TDA hybrid approach for classifying BCC vs non-BCC dermoscopic lesion images. It exploits color space information to calculate persistence homology topological features for skin lesion images and includes topological features from a clinical biomarker for BCC, telangiectasia. For the deep learning model, a state-of-the-art pretrained model, EfficientNet-B5, was chosen. Combining the DL and TDA features, the hybrid DL-TDA model outperforms EfficientNet-B5 as well as other convolution neural network–based pretrained models. State-of-the-art accuracy and precision were achieved over a larger dataset publicly available at [40] than in previous studies. With the inclusion of the telangiectasia features and the subsequent improvements in the final classification result, a clinically explainable aspect of the diagnosis was demonstrated that can be extended to other biomarkers.

In the future, this research can be extended by incorporating a broader range of clinical features into this hybrid model. Additionally, statistical techniques, such as ANOVA with suitable post hoc tests, can be applied to address significant differences.

## Data Availability

The telangiectasia masks and the corresponding BCC images used in this study are publicly available at: 10.5281/zenodo.7709824.
